# CD56^bright^ natural killer cells preferentially kill proliferating CD4^+^ T cells

**DOI:** 10.1093/discim/kyad012

**Published:** 2023-08-11

**Authors:** Mercede Lee, Charles J M Bell, Arcadio Rubio Garcia, Leila Godfrey, Marcin Pekalski, Linda S Wicker, John A Todd, Ricardo C Ferreira

**Affiliations:** JDRF/Wellcome Diabetes and Inflammation Laboratory, Wellcome Centre for Human Genetics, Nuffield Department of Medicine, NIHR Oxford Biomedical Research Centre, University of Oxford, Oxford, UK; JDRF/Wellcome Diabetes and Inflammation Laboratory, Wellcome Centre for Human Genetics, Nuffield Department of Medicine, NIHR Oxford Biomedical Research Centre, University of Oxford, Oxford, UK; JDRF/Wellcome Diabetes and Inflammation Laboratory, Wellcome Centre for Human Genetics, Nuffield Department of Medicine, NIHR Oxford Biomedical Research Centre, University of Oxford, Oxford, UK; JDRF/Wellcome Diabetes and Inflammation Laboratory, Wellcome Centre for Human Genetics, Nuffield Department of Medicine, NIHR Oxford Biomedical Research Centre, University of Oxford, Oxford, UK; JDRF/Wellcome Diabetes and Inflammation Laboratory, Wellcome Centre for Human Genetics, Nuffield Department of Medicine, NIHR Oxford Biomedical Research Centre, University of Oxford, Oxford, UK; JDRF/Wellcome Diabetes and Inflammation Laboratory, Wellcome Centre for Human Genetics, Nuffield Department of Medicine, NIHR Oxford Biomedical Research Centre, University of Oxford, Oxford, UK; JDRF/Wellcome Diabetes and Inflammation Laboratory, Wellcome Centre for Human Genetics, Nuffield Department of Medicine, NIHR Oxford Biomedical Research Centre, University of Oxford, Oxford, UK; JDRF/Wellcome Diabetes and Inflammation Laboratory, Wellcome Centre for Human Genetics, Nuffield Department of Medicine, NIHR Oxford Biomedical Research Centre, University of Oxford, Oxford, UK

**Keywords:** Natural killer (NK) cells, CD56^br^ NK cells, CD4^+^ T cells, in vitro NK killing assay, low-dose IL-2 immunotherapy

## Abstract

Human CD56^br^ natural killer (NK) cells represent a small subset of CD56^+^ NK cells in circulation and are largely tissue-resident. The frequency and number of CD56^br^ NK cells in blood has been shown to increase following administration of low-dose IL-2 (LD-IL2), a therapy aimed to specifically expand CD4^+^ regulatory T cells (Tregs). Given the potential clinical application of LD-IL-2 immunotherapy across several immune diseases, including the autoimmune disease type 1 diabetes, a better understanding of the functional consequences of this expansion is urgently needed. In this study, we developed an *in vitro* co-culture assay with activated CD4^+^ T cells to measure NK cell killing efficiency. We show that CD56^br^ and CD56^dim^ NK cells show similar efficiency at killing activated CD4^+^ conventional T (Tconv) and Treg cell subsets. However, in contrast to CD56^dim^ cells, CD56^br^ NK cells preferentially target highly proliferative cells. We hypothesize that CD56^br^ NK cells have an immunoregulatory role through the elimination of proliferating autoreactive CD4^+^ Tconv cells that have escaped Treg suppression. These results have implications for the interpretation of current and future trials of LD-IL-2 by providing evidence for a new, possibly beneficial immunomodulatory mechanism of LD-IL-2-expanded CD56^br^ NK cells.

## Introduction

Natural killer (NK) cells represent a population of innate immune cells that have evolved to mediate their effector function through the acquisition of a cytotoxic profile, while avoiding tissue damage [1]. Although typically associated with a multitude of effector functions, including tumour cell recognition and anti-viral immunity, NK cells have also been more recently shown to display immunomodulatory functions through the regulation of T cell responses [2–6]. In humans, circulating CD56^+^ NK cells can be differentiated into two main subtypes based on the expression of CD56 and CD16: a rare CD56^br^ CD16^–/low^ (CD56^br^) subset and a more abundant CD56^dim^ CD16^+^ (CD56^dim^) subset, usually representing > 90% of circulating NK cells. In contrast to blood, CD56^br^ NK cells are predominantly found in tissues, where they represent the majority of tissue-resident NK cells [7]. While it is known that CD56^+^ NK cells develop from CD34^+^ haematopoietic stem cells, there is conflicting data on whether CD56^br^ NK cells are precursors of CD56^dim^ NK cells or if they constitute an independent NK cell lineage [8, 9]. More recently, the application of multi-parametric multiomics technologies have revealed a more complex picture of the phenotypic heterogeneity of NK cell subsets in blood and in tissues [10–15].

In addition to CD4^+^ regulatory T cells (Tregs), circulating CD56^br^ NK cells expand greatly in response to low-dose interleukin-2 (LD-IL-2) treatment [16–19]. While both CD56^+^ NK cell subsets express high levels of the IL-2 receptor β chain (CD122), the increased sensitivity of CD56^br^ NK cells to LD-IL-2 is mediated by the concomitant increased expression of the high-affinity α chain (CD25) [18]. The initial therapeutic success and ongoing clinical trials of LD-IL-2 immunotherapy in a variety of autoimmune and inflammatory diseases [20, 21] therefore provide an important rationale to better understand the potential contribution of CD56^br^ NK cells in the mechanism of action of LD-IL-2 immunotherapy. However, the exact function of CD56^br^ NK cells is still poorly understood in the context of their expansion by LD-IL-2 and the therapeutic potential of this drug. One of the main challenges is the scarcity of these cells in blood, which strongly limits the access to the cell numbers required for the development of robust cellular assays. Here, we detail the development of an *in vitro* NK:CD4^+^ T cell co-culture assay. We demonstrate that in contrast to CD56^dim^ NK cells, CD56^br^ NK cells preferentially kill the more highly proliferative CD4^+^ T cells, suggesting a regulatory function in the elimination of pathogenic proliferating T cells in inflamed tissues.

## Methods

### Subjects

A total of 29 healthy study participants were recruited from the Oxford BioBank. Approximately 50 ml of peripheral venous blood was collected in Ethylenediaminetetraacetic acid tubes from each donor. All samples and information were collected with written and signed informed consent. The study conformed to the principles of the Helsinki Declaration and Good Clinical Practice and was approved by the East of England—Cambridgeshire and Hertfordshire research ethics committee (05/Q0106/20).

### Cell preparation

For assays using freshly isolated peripheral blood mononuclear cells (PBMCs; *n* = 14 donors), cells were isolated from human peripheral blood samples within 2 h of venepuncture. Whole blood samples were diluted at a 1:1 ratio with Roswell Park Memorial Institute (RPMI) 1640 medium (Sigma) and then gently layered over density gradient medium Lymphoprep™ (STEMcell). PBMCs were isolated by centrifugation at 800 g for 20 min. PBMCs were washed twice in phosphate-buffered saline (PBS; Gibco) with 0.2% bovine serum albumin (BSA; Miltenyi Biotec) and re-suspended in 10 ml PBS + 0.2% BSA for cell counting.

For assays using cryopreserved cells (*n* = 15 donors), PBMCs were re-suspended at a concentration of 10^6^ cells/ml in CryoStor. Samples were incubated overnight at −80°C in a cryogenic storage container (CoolCell™, Corning) and then stored in liquid nitrogen until the day of the assay. Samples were then thawed at 37°C, re-suspended drop-by-drop in RPMI with 1% heat-inactivated, filtered human AB serum (AB HS; Sigma), and washed twice with PBS + 0.2% BSA.

### Cell staining and fluorescence-activated cell sorting (FACS)

To isolate the CD4^+^ T cell subsets for our assays, approximately 5% of the PBMCs (T cell fraction) were washed with PBS only and then incubated for 10 min at 37°C with cell proliferation dye eFluor™ 450 (Invitrogen) at a concentration of 10 µM. Following incubation, the cell proliferation dye was quenched with ice-cold PBS + 10% BSA and incubated on ice for a further 5 min, then washed with PBS + 0.2% BSA. The remaining 95% of PBMCs (NK cell fraction) were dedicated for the isolation of the CD56^+^ NK subsets and were not incubated with the cell proliferation dye prior to cell sorting.

Both cell fractions were then incubated with Brilliant Stain Buffer (BD Biosciences) and incubated for 30 min at 4°C with the following fluorochrome-conjugated antibodies: CD3-BUV737, CD4-BUV395, CD25-PE (BD Biosciences), CD127-PE Cy7 (eBioscience), CD56-BV711, and CD8-AF700 (Biolegend). PBMCs were then washed and re-suspended in PBS + 0.2% BSA for cell sorting on a BD FACSAria™ Fusion cell sorter (BD Biosciences). The following cell subsets were sorted: CD3^+^CD4^+^CD127^+/low^CD25^–/low^ [CD4^+^ conventional T (Tconv)] and CD3^+^CD4^+^ CD127^low^CD25^hi^ (CD4^+^ Tregs) from the T cell fraction (stained with proliferation dye); and CD3^–^CD56^hi^CD127^+/low^ (CD56^br^ NK) and CD3^–^CD56^low^CD127^–^ (CD56^dim^ NK) from the NK cell fraction (proliferation dye-negative). Post-sorting cell purity rates were found to be > 94% and > 97% for the NK and T cell subsets, respectively.

### Cell culture

All cell subsets were washed with PBS + 0.2% BSA and then re-suspended in master media [RPMI + 100U penicillin + 100 µg streptomycin + 2 mM l-Glutamine (Gibco) + 5% AB HS] + 100 IU/ml IL-2 (Proleukin®/aldesleukin; Novartis) at a target concentration of 5 × 10^5^ cells/ml and 100 µl (50 000 cells) were seeded in a 96 well U-bottom plate (Greiner). The main limitation for the practical implementation of these *in vitro* co-culture assays is the number of CD56^br^ NK cells that can be sorted from each donor, as their frequency is variable between donors and they represent only a small fraction of the total NK cells. This was particularly challenging for cryopreserved samples, where we achieved a median of 41 198 [95% confidence interval (CI) 24 047–73 024] sorted CD56^br^ NK cells. By contrast, we obtained much higher numbers of sorted CD56^br^ NK cells from freshly isolated PBMCs (median = 122 700; 95% CI 69 416–210 000). We, therefore, opted to use only cells sorted from freshly isolated PBMCs to set up the killing assays described in this study.

CD4^+^ T cells were activated using αCD3/CD28 beads (Gibco) at a ratio of 1 bead: 3 T cells in addition to IL-2 (100 IU/ml), whereas activation of NK subsets only required 100 IU/ml IL-2. Cell subsets were cultured separately for up to 3.5 days *in vitro*. To assess the proliferation dynamics of both CD4^+^ T and CD56^+^ NK cell subsets, cells were processed as described above using 10 × 10^6^ cryopreserved PBMCs from four independent healthy donors and stained with the proliferation dye eFluor™ 450 (Invitrogen), before culture. CD4^+^ T cells were cultured as described above and CD56^+^ NK cells were cultured either in the presence or absence of 100 IU/ml IL-2. All cells were harvested at days 3 and 7 to assess proliferation on BD LSRFortessa™ (BD Biosciences).

### NK killing assay

At 2.5 or 3.5 days of *in vitro* cell culture, CD4^+^ T and CD56^+^ NK cell subsets were incubated for 20 min at room temperature with CD4-BUV395 (BD) and CD56-BV711 (BioLegend), respectively. CD4^+^ T cell subsets were then washed and co-cultured with the respective CD56^+^ NK cell subset. Before co-culture, each CD4^+^ T and CD56^+^ NK cell subsets were carefully counted using a haemocytometer to achieve the intended 5 NK: 1 T cell ratio. Due to the limiting number of NK cells, the number of cells used for co-culture in each assay were determined by the available NK cells. Sufficient activation of CD56^br^ NK cells was achieved for most of the donors, except for one donor pair, where we were only able to use 5000 CD56^br^ NK cells for the co-culture assays. We obtained much higher numbers of CD56^br^ NK for all other donor pairs (median = 28 000 cells, 95% CI 13 000–67 500). Co-cultures were incubated for 5 h, at 37°C in master media with 100 IU/ml IL-2. Immediately before cells were analysed, co-cultures were incubated in the dark with fluorescent cell viability dye (DRAQ7; BioLegend) for 15 min at room temperature. Each well was rinsed with PBS + 0.2% BSA and re-suspended in a final volume of 300 µl for analysis on BD LSR Fortessa™ (BD Biosciences). To ensure no dead T cells were removed from the analysis—hence skewing the assessment of the frequency of specific killing—we analysed all acquired cells, without prior forward scatter (FSC) versus side scatter (SSC) exclusion. CD4^+^ T were discriminated from CD56^+^ NK cells based on the expression of CD4 and proliferation dye. Frequency of specific killing was then calculated by subtracting the percentage of background spontaneous cell death, assessed in both CD4^+^ Tconv (median = 8.4%, 95% CI 4.9–19.5%) and Tregs (median = 9.6%, 95% CI 6.8–13.0%) from each donor incubated in the absence of CD56^+^ NK cells.

To calculate the relative frequency of specific killing according to the proliferative stage of the responder T cells, we have defined “highly proliferative” T cells, as CD4^+^ Tconvs and Tregs having undergone ≥2 proliferation cycles at day 2.5 or ≥3 proliferation cycles at day 3.5. The different thresholds used at day 2.5 and day 3.5 were set to account for the lower proliferation dynamics of CD4^+^ T cells at day 2.5 compared to day 3.5. The frequency of specific killing in highly proliferative and lowly proliferative cells was then assessed by determining the percentage of dead responder CD4^+^ T cells in each respective population, after subtracting the percentage of background spontaneous cell death (assessed in responder T cells cultured in the absence of CD56^+^ NK cells) in both the high (Tconv: median = 12.6%, 95% CI 6.4–20.1%; Treg: median = 10.7%, 95% CI 7.7–15.0%) and low (Tconv: median = 7.6%, 95% CI 3.6–17.0%; Treg: median = 10.4%, 95% CI 6.4–13.3%) proliferation groups.

### Data analysis

The software package FlowJo (BD Biosciences, V10.8.1) was used for the analysis of the FCS files obtained from the flow cytometer. FlowJo’s proliferation modelling software was used to analyse the proliferative capacity of cell subsets. Proliferative capacity was calculated using both the division and proliferation index (PI) methods implemented in FlowJo. The division index (DI) models proliferation for the whole population, while the PI, only models the cells that have initiated cell division. Differences in the proliferative capacity of cell subsets was compared using two-tailed paired t-tests.

Statistical analyses of NK killing assay data were performed by Bayesian inference on a Beta regression model [22], with cell type and sample pair as predictor variables, and killing proportion as response variable. We depict posterior distributions of killing proportion means for each group and fold changes across groups. As a summary of posterior distributions, we report the median and a 95% credible interval. As a measure of uncertainty, we refer to the false sign rate (FSR) [23]. This quantity denotes the support of the posterior distribution for an effect with a sign different than the median, i.e. the probability that killing proportions are higher on the group whose killing proportion is estimated to be lower. Posterior distributions were simulated using a No-U-Turn sampler (Stan, v2.31) [24] with default parameters and four chains that drew 2500 samples each during warmup, followed by another 2500 samples for estimation. Prior distributions were set to default weakly informative choices for regularization and stabilization.

## Results

### Assessing the proliferative capacity of in vitro stimulated immune cell subsets

The investigation of the functional relevance of NK cell subsets has been hampered by the lack of suitable *in vitro* assays. Currently, the most widely used NK functional assays include co-culture assays, aiming at quantifying NK killing of the target T cells subset. Previous studies have shown that both the NK cells and target T cells require appropriate activation for efficient autologous killing to occur *in vitro* [25]. One of the main limitations for the development of more robust *in vitro* assays is the availability of sufficient cell numbers, particularly for the rare subset of circulating CD56^br^ NK cells. To overcome this limitation, we aimed to establish an *in vitro* NK cell killing assay that minimizes the required number of CD56^+^ NK and CD4^+^ T cell subsets sorted from PBMCs.

**Figure 1:**
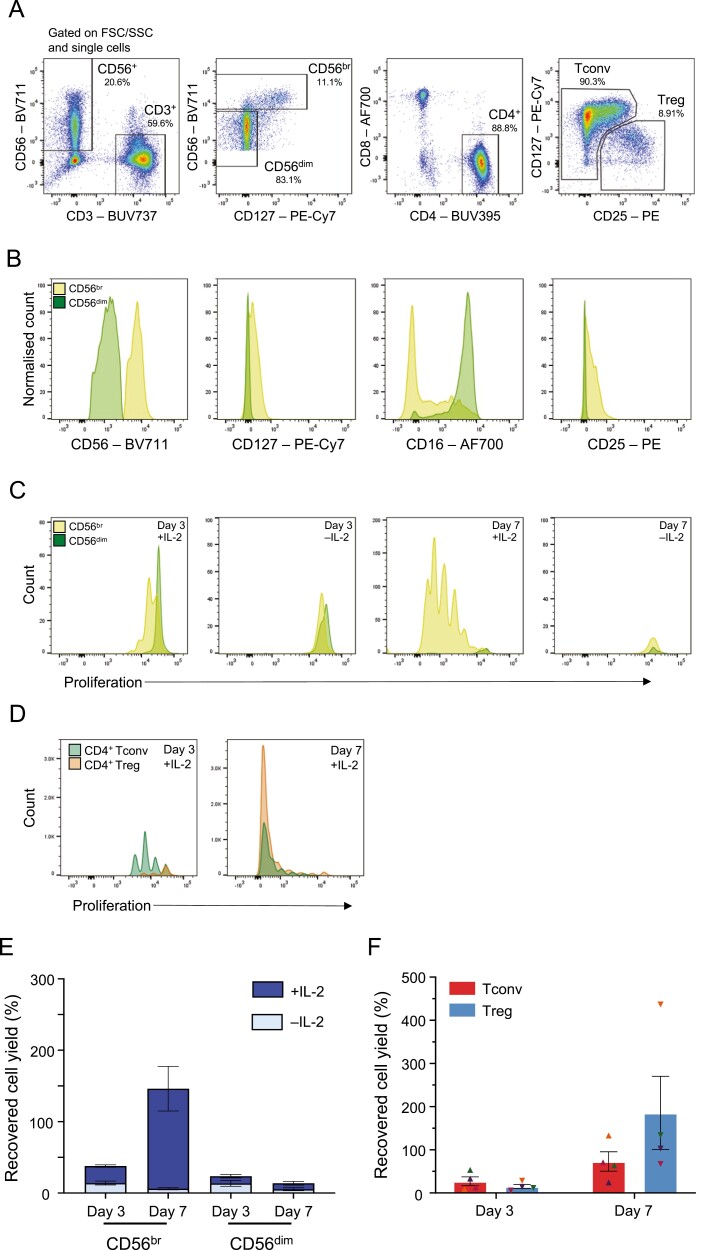
Assessing the proliferative capacity of the CD56^+^ NK cell subsets *in vitro*. (A) Sorting strategy for the isolation of the four assessed immune cell subsets: (i) CD3^–^CD56^br^ NK cells (CD56^br^ NK); (ii) CD3^–^CD56^dim^ NK cells (CD56^dim^ NK); (iii) CD3^+^CD4^+^CD25^–/low^ (Tconv); and (iv) CD3^+^CD4^+^CD127^low^CD25^hi^ (Treg). Plots represent an illustrative example from the sorting of cryopreserved PBMCs from four healthy donors. (B) Representative histograms depicting the expression of CD56, CD127, CD16, and CD25 in sorted CD56^br^ (light green) and CD56^dim^ (dark green) NK cells at day 0. (C) Representative histograms depicting the proliferative capacity of CD56^br^ (light green) and CD56^dim^ (dark green) NK cells after 3 (left panels) or 7 (right panels) days of culture in the presence or absence of 100 IU/ml IL-2. (D) Representative histograms depicting the proliferative capacity of CD4^+^ Tconvs and Tregs after 3 (left panel) or 7 (right panel) days of culture in the presence of 100 IU/ml IL-2 and TCR stimulation with αCD3/CD28 beads (1 bead: 3 T cells). (E and F) Bar plots depict the distribution (mean ± SEM) of the recovered cell yields (defined as the percentage of plated cells harvested after the initial 3 or 7 days *in vitro* culture period) in both CD56^+^ NK cell (E) and CD4^+^ T cell (F) subsets. CD4^+^ Tconvs (depicted by triangles) and Tregs (depicted by inverted triangles) isolated from the same donor are depicted using the same colour.

Initially, we explored the use of cryopreserved PBMCs to set up the *in vitro* assay. We flow-sorted CD56^br^ NK, CD56^dim^ NK, CD4^+^CD25^–/low^ Tconv, and CD4^+^ Treg cell subsets (Fig. 1A) and cultured both TCR-stimulated T cell and NK cell subsets for a period of 3 or 7 days *in vitro* in the presence of 100 IU/ml IL-2. As expected, sorted CD56^br^ and CD56^dim^ NK cell subsets displayed distinct phenotypic profiles, as evidenced by the expression of key markers, such as CD56, CD127, CD16, and CD25 (Fig. 1B). A feature of our assay was the ability to assess the proliferation status of the different cell subsets at the single-cell level using an intracellular cell proliferation dye, resulting in distinct proliferation peaks. To investigate the dynamics of cell proliferation in both CD56^+^ NK cell and TCR-stimulated CD4^+^ T cell subsets, we initially assessed the proliferative capacity of both populations at days 3 and 7 of culture to determine the optimal duration of the initial stimulation period. Notably, CD56^+^ NK cell proliferation *in vitro* was mostly restricted to the CD56^br^ NK cells and was strictly dependent on the presence of IL-2 in culture (Fig. 1C). In contrast to CD56^+^ NK cells, both T cell subsets demonstrated strong *in vitro* proliferative capacity following stimulation with αCD3/CD28 beads (1 bead: 3 T cells) and IL-2 (Fig. 1D). At day 7, the majority of CD4^+^ Tconv and Tregs in culture had undergone over four-division cycles suggesting excessive proliferation that may compromise the potential to assess cell subset-specific functional differences.

In agreement with their lower proliferative capacity, we obtained much lower cell recovery (defined as the percentage of the initial plated cells that were harvested) for the CD56^+^ NK cell subsets compared to the T cell subsets after 7 days of culture (Fig. 1E and F). This was particularly pronounced in the CD56^dim^ NK cell subset at day 7, where we obtained only a marginal 8.41% median cell recovery (Fig. 1E). These numbers reflect the lack of proliferative capacity of CD56^dim^ NK cells in this *in vitro* model and demonstrate the reduced viability of CD56^dim^ NK cells *in vitro*, thereby justifying the use of an initial 3-day stimulation period to set up our co-culture assay. Importantly, the low cell yields obtained for the CD56^+^ NK cell subsets using cryopreserved PBMCs compromised the technical integrity of the co-culture assays, leading to insufficient numbers of target T cells being plated for the assay (at an intended ratio of 5 NK cells: 1 T cell) to robustly estimate the proportion of T cell death after co-culture. To mitigate this issue, we subsequently opted to perform this assay using freshly isolated PBMCs, which yielded much higher numbers of the sorted NK cell subsets, most notably of the limiting CD56^br^ subset.

### Development of an in vitro NK cell-killing assay

**Figure 2:**
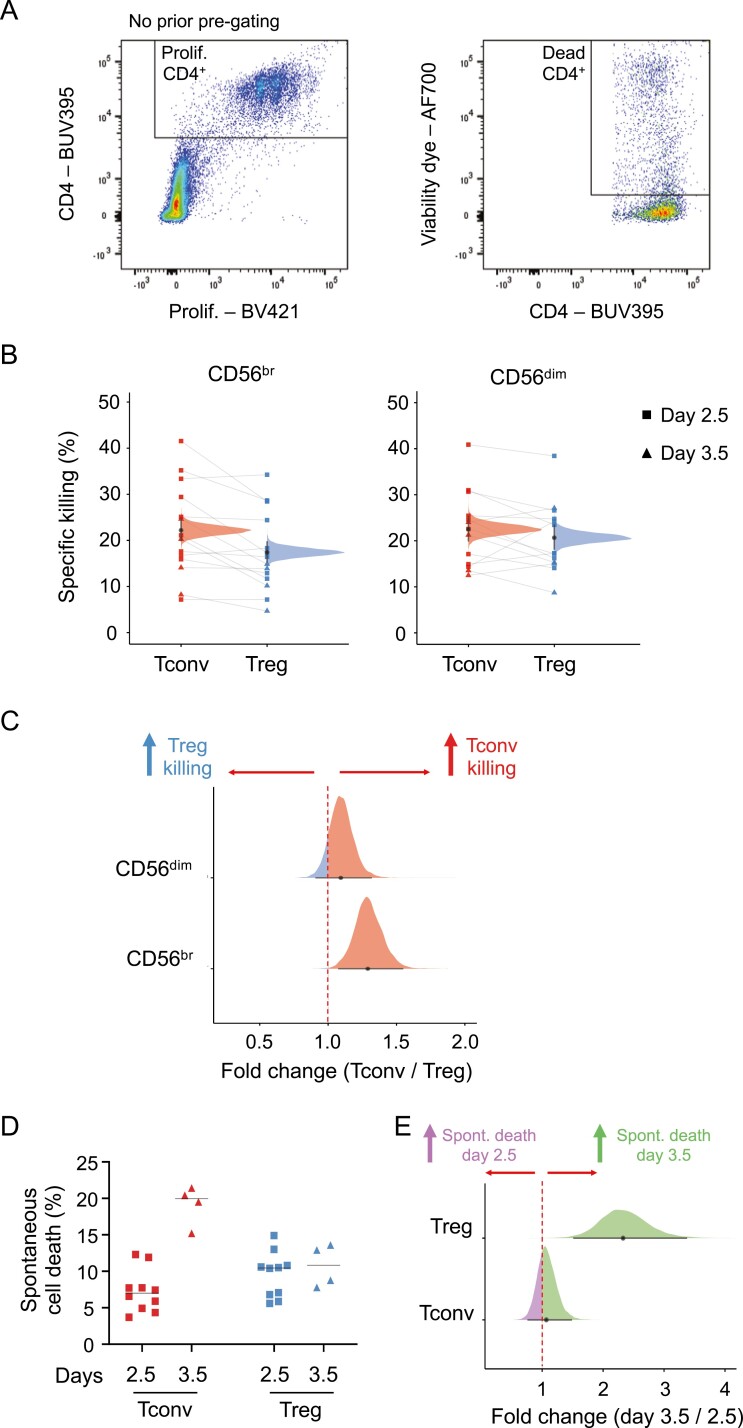
Development of a CD56^br^ and CD56^dim^ NK cell *in vitro* killing assay. (A) Gating strategy for the quantification of dead responder T cells from the *in vitro* killing assays. Killing assays were performed using T and NK cells sorted from freshly isolated PBMCs from 14 healthy donors. (B) Scatter plot depicts the frequency of specific killing of CD4^+^ Tconvs (red) and Tregs (blue). Frequency of specific killing was determined as the relative proportion of dead responder T cells after co-culture with CD56^br^ (left panel) and CD56^dim^ (right panel) NK cells, after subtracting the relative proportion of background spontaneous cell death, estimated in both responder T cell subsets (Tconvs and Tregs) incubated in the absence of NK cells (see Methods). Individuals where the initial stimulation period was 2.5 days (*n* = 10) or 3.5 days (*n* = 4) are depicted by squares or triangles, respectively. Densities depict the mean posterior frequency of cell death values in each group estimated by beta regression, with an annotation for the median and a 95% credible interval. CD4^+^ Tconv and Treg cells isolated from the same donor are identified by a connecting line. (C) Densities depict the effect size distribution (measured as fold change) for the observed relative rates of specific killing in CD4^+^ Tconv (red) and Treg (blue) by CD56^br^ (bottom panel) and CD56^dim^ (top panel) NK cells. (D) Scatter plots depicts the percentage of spontaneous cell death of CD4^+^ Tconv (red) and Treg (blue) cells. The frequency of spontaneous cell death was assessed in each donor by culturing each T cell subset in the absence of CD56^+^ NK cells. Data were stratified according to whether the CD4^+^ T cells subsets were stimulation for an initial period of 2.5 (depicted by squares) or 3.5 (depicted by triangles) days. (E) Densities depict the effect size distribution (measured as fold change) for the observed relative rates of spontaneous cell death at days 2.5 and 3.5 in CD4^+^ Tconv (bottom panel) and Treg (top panel) cells.

Having established the duration of the initial activation period, we then proceeded to perform the killing assays using sorted NK and T cells from freshly isolated PBMCs from seven pairs of donors. After an initial activation period of up to 3.5 days, activated NK and T cells were then co-cultured at a target ratio of five effector cells (CD56^+^ NK cell subset) to one target cell (CD4^+^ T cell subset). The proportion of dead T cells was then assessed by FACS after a 4 h co-culture period, and a frequency of specific killing was calculated by subtracting the background frequency of spontaneous cell death obtained in each CD4^+^ T cell subset incubated in the absence of CD56^+^ NK cells (Fig. 2A and Methods). Both CD56^br^ and CD56^dim^ NK cells displayed similar capacity to kill autologous CD4^+^ T cells in our *in vitro* assay. Of note, CD56^br^ NK cells displayed a small increased frequency of specific killing for CD4^+^ Tconv (median posterior probability (PP) = 22.2%, 95% credible interval = 20.2–24.4%) compared to CD4^+^ Treg (median PP = 17.4%, 95% credible interval = 15.5–19.5%) cells (FSR = 0.2%; Fig. 2B and C). In contrast, we observed no difference in the frequency of specific killing of CD4^+^ Tconv (median PP = 22.5%, 95% credible interval = 20.3–25.0%) or Treg (median PP = 20.7%, 95% credible interval = 18.5–23.1%) cells by CD56^dim^ NK cells (1.09 fold change, FSR = 12%; Fig. 2B and C).

To assess whether the observed differences in the frequencies of specific killing were partly due to increased apoptosis in more activated cells, we next compared the frequency of spontaneous cell death between the two responder T cell subsets. CD4^+^ Tconv cells stimulated for 3.5 days showed a 2.33-fold increased frequency (FSR = 0.04%; Fig. 2D and E) of spontaneous cell death compared with 2.5 days of stimulation (median PP = 7.0%, 95% credible interval = 5.5–8.9% and 16.2%, 95% credible interval = 12.5–20.2% and at 2.5 and 3.5 days, respectively). In contrast, no discernible differences could be observed in CD4^+^ Treg cells (median PP = 8.6% and 9.7% at 2.5 and 3.5 days, respectively; Fig. 2D and E). Nevertheless, we note that the increased rate of spontaneous cell death in more activated CD4^+^ T conv cells did not cause noticeable differences in the frequency of spontaneous cell death between the two responder T cell subsets (Fig. 2D and E). These data, therefore, indicate that the reported differences in the frequency of specific killing of CD4^+^ Tconvs and Tregs by CD56^br^ NK cells are not simply caused by differences in the rate of spontaneous cell death between the two subsets.

### CD4^+^ Tconv and Treg cells display different proliferation dynamics in vitro

**Figure 3:**
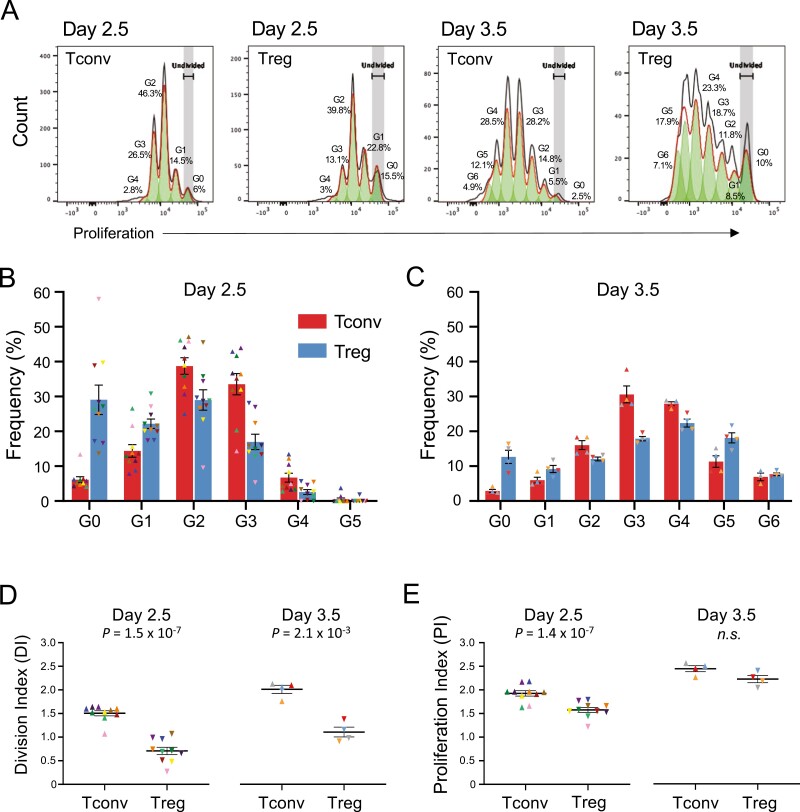
Assessing the proliferative capacity of CD4^+^ T cells *in vitro*. (A) Representative histograms depicting the proliferative capacity of CD4^+^ Tconv and Treg cells after 2.5 or 3.5 days of *in vitro* culture in the presence of 100 IU/ml IL-2 and TCR stimulation with αCD3/CD28 beads (1 bead: 3 T cells). The respective cell generation (G_n_) are shown in each histogram. G_0_ represents undivided CD4^+^ T cells. Data were generated from freshly isolated PBMCs from 14 healthy donors (*n* = 10 and *n* = 4 for day 2.5 and day 3.5, respectively). (B) Histograms depict the frequency (mean ± SEM) of CD4^+^ Tconv (red) and Treg (blue) cells at each generation of cell division after 2.5 (B) and 3.5 (C) days of stimulation with αCD3/CD28 beads and 100 IU/ml IL-2, followed by an additional 4 h of co-culture in the presence or absence of the respective CD56^+^ NK cell subset. The reported frequencies of proliferating CD4^+^ Tconvs and Tregs for each donor at each generation represent the average of the three co-culture conditions assessed in this study: (i) co-culture with CD56^br^ NK cells; (ii) co-culture with CD56^dim^ NK cells; and (iii) cultured alone in the absence of NK cells (background control to assess spontaneous cell death). The T cell proliferation rates were not affected by the different co-culture conditions, and were therefore averaged to increase the number of T cells assessed. CD4^+^ Tconvs (depicted by triangles) and Tregs (depicted by inverted triangles) isolated from the same donor are depicted using the same colour. (D) Scatter plots depict the distribution (mean ± SEM) of the observed DI for CD4^+^ Tconv and Treg cells after co-culture at 2.5 (left panel) or 3.5 (right panel) days of stimulation. (E) Same as for D, but depicting the PI of the cells. *P* values were calculated using two-tailed paired t-tests. DI denotes the average number of cell divisions undergone by a cell in the original population and PI denotes the average number of divisions among the cells that have divided at least once. n.s., *P* value not significant.

To further investigate the causes leading to the slight preferential killing of CD4^+^ Tconvs by CD56^br^ NK cells, we next investigated whether intrinsic differences in the response of the two CD4^+^ T cell subsets to stimulation in our *in vitro* model could explain this result. We first assessed whether differences in the *in vitro* proliferation dynamics of CD4^+^ Tconvs and Tregs stimulated with αCD3/CD28 beads and IL-2 could contribute for this result (Fig. 3A). We note that because only the CD4^+^ T cell subsets were stained with the intracellular cell proliferation dye in our NK cell killing assay, the combination of the proliferation dye and CD4 antibody staining allowed a very clear distinction of the CD4^+^ T and the CD56^+^ NK cell subsets following co-culture. This stratification of the responder T cell subsets allowed us to compare the dynamics of response to αCD3/CD28 stimulation in both CD4^+^ Tconv and Treg subsets. One notable difference between the T cell subsets was associated with the timing of initial response to stimulation, and entry into cell cycle. CD4^+^ Tconvs showed a more rapid response to stimulation, as illustrated by the substantial increased proportion of cells having divided at least once by day 2.5 (94% and 71% in CD4^+^ Tconvs and Tregs, respectively; Fig. 3B). In contrast, one day later (day 3.5 post-stimulation), this difference was much less pronounced, with a mean of 97% and 87% of CD4^+^ Tconvs and Tregs, respectively, having divided at least once (Fig. 3C). Another notable difference between CD4^+^ Tconv and Treg proliferation dynamics was the range of proliferation cycles achieved by both subsets. At day 2.5 post-stimulation only 0.51% and 0.19% of CD4^+^ Tconvs and Tregs, respectively, had already achieved the fifth proliferation cycle (G5; Fig. 3B). However, by day 3.5 we observed a striking difference in the frequency of cells that had undergone five or more cell cycles, with CD4^+^ Tregs showing a substantial increased proportion of highly proliferative cells compared to Tconvs (25.81% and 16.52%, respectively; Fig. 3C).

To further understand the differences in the proliferation dynamics of the CD4^+^ Tconv and Treg subsets, we then modelled their proliferation rate using two different methods, namely the DI (average number of cell divisions undergone by a cell in the original population) and the PI (average number of divisions among the cells that have divided at least once). At both days 2.5 and 3.5 post-stimulation, we observed a consistently higher DI in CD4^+^ Tconvs compared to Tregs (*P* = 1.5 × 10^−7^ and *P* = 2.1 × 10^−3^, respectively; Fig. 3D), indicating that on average more CD4^+^ Tconvs have divided at least once compared to their respective Treg counterparts. In contrast to the DI, while CD4^+^ Tconvs also displayed an increased PI compared to Tregs at day 2.5 (*P* = 1.4 × 10^−7^), this difference was nullified at day 3.5 (Fig. 3E), indicating that although less Treg cells are actively proliferating, on average the proliferating fraction has undergone more cell cycles than the proliferating Tconv counterparts. Taken together these results support different dynamics of response to αCD3/CD28 stimulation *in vitro* by CD4^+^ Tconvs and Tregs, with Tconvs showing increased sensitivity to initial stimulation, but displaying a more limited potential to proliferate compared to Tregs.

### CD56^br^ NK cells preferentially target highly proliferative CD4^+^ T cells

**Figure 4:**
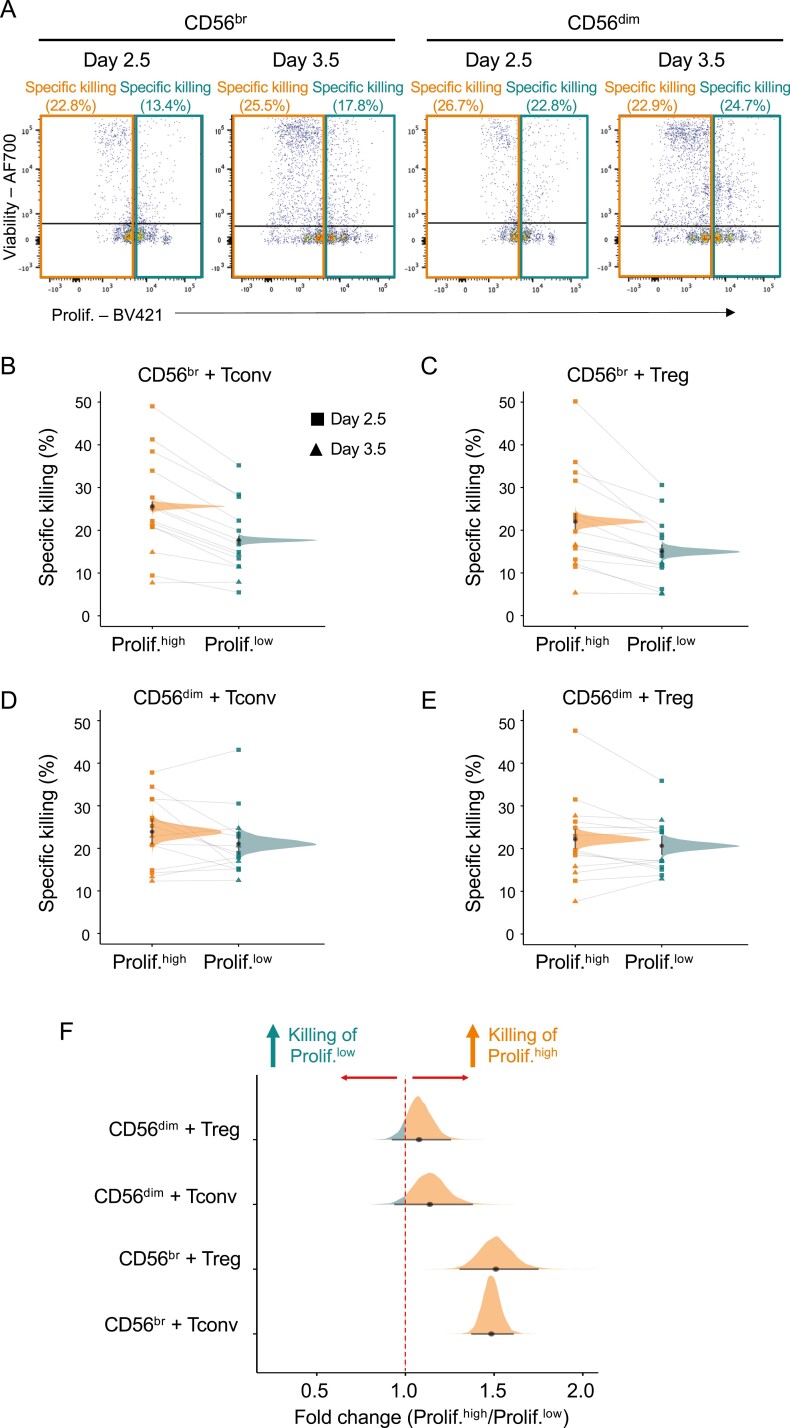
CD56^br^ NK cells preferentially kill highly proliferative CD4^+^ T cell subsets. (A) Representative dot plots depicting the gating strategy used to define the frequency of T cell death among highly (prolif.^high^; orange) and lowly (prolif.^low^; teal) proliferative CD4^+^ T cells co-cultured with CD56^br^ (left panels) and CD56^dim^ (right panels)NK cells. The relative frequency of cell death was calculated in each proliferative group and takes the background levels of spontaneous CD4^+^ T cell death (CD4^+^ T cells co-cultured without NK cells) into account (see Methods). Data were generated from freshly isolated PBMCs from 14 healthy donors for day 2.5 (*n* = 10; squares) and day 3.5 (*n* = 4; triangles), respectively. (B and C) Scatter plots depict the relative frequency of cell death in CD4^+^ Tconv (B) and Treg (C) cells from the prolif.^high^ and prolif.^low^ proliferation groups after co-culture with CD56^br^ NK cells. (D and E) Same as B and C but for CD4^+^ Tconvs (D) and Tregs (E) co-cultured with CD56^dim^ NK cells. Densities depict the mean posterior frequency of cell death values in each group estimated by Beta regression, with an annotation for the median and a 95% credible interval. (F) Densities depict the effect size distribution (measured as fold change) for the observed relative rates of specific killing in highly and lowly proliferative for each of the CD4^+^ responder T cell subsets by either CD56^br^ (top panels) or CD56^dim^ (bottom panels) NK cells.

In many autoimmune diseases, CD4^+^ T cells have been implicated in mediating aspects of inflammation. Therefore, the body must regulate the number of CD4^+^ T cells to maintain tolerance. Given the high sensitivity of our assay to specifically interrogate T cell proliferation state, we next investigated whether CD56^br^ NK cells and/or CD56^dim^ NK cells could be differentially targeting T cells according to their proliferative state. Given the observed dynamics of T cell proliferation under our assay conditions, we have defined in this study highly proliferative CD4^+^ Tconvs and Tregs as cells having undergone two or more proliferation cycles at day 2.5 and more than three proliferative cycles at day 3.5 (Fig. 4A). We then assessed the relative frequency of dead responder CD4^+^ T cells in each respective proliferative group. We found that CD56^br^ NK cells differentially killed target T cells according to their proliferative state.

Both CD56^br^ and CD56^dim^ NK cell subsets displayed similar frequencies of specific killing of the autologous CD4^+^ T cell subsets irrespective of their proliferative state (Fig. 4B–E). Notably, highly proliferative CD4^+^ Tconv and Tregs were preferentially killed by CD56^br^ NK cells (median PP = 25.6%, 95% credible interval = 24.6–26.6% and 22.0%, 95%credible interval = 20.5–23.7%, respectively) compared to their respective less proliferative counterparts (median = 17.7%, 95%credible interval 16.8–18.6% and 15.0%, 95% credible interval 13.6–16.4%, respectively; Fig. 4B and C). These differences corresponded to a median fold-change increase of 1.49 and 1.51 (FSR < 0.001%) in the frequency of specific killing of highly proliferative CD4^+^ Tconv and Treg cells, respectively (Fig. 4F). We note that the increased preference of CD56^br^ NK cells to kill proliferative autologous T cells can also explain the observed small increased relative killing of CD4^+^ Tconvs compared to Tregs (Fig. 2B), which could be attributed to the increased proliferative state of Tconvs compared to Tregs. In contrast to CD56^br^ NK cells, CD56^dim^ NK cells showed no preferential killing of either responder T cell subset according to their respective proliferative state (median 0.96 and 0.94 fold change, respectively, and FSR > 5% in both cases; Fig. 4E and F), suggesting that the observed preferential killing of highly proliferative T cells in our *in vitro* model is a specific feature of CD56^br^ NK cells.

## Discussion

The characterization of NK cell biology has been hampered by technical limitations with *in vitro* assay development, ranging from limited cell numbers in peripheral blood to the variability in assay conditions (e.g. the combination of cytokines used in each specific assay). In this study, through the development of a NK killing assay, we demonstrated the specific consequences of these limitations and the necessity to distinguish between the CD56^br^ and CD56^dim^ NK cell subsets. In agreement with their terminally differentiated phenotype [13], CD56^dim^ NK cells display very limited proliferative capacity *in vitro* when stimulated with IL-2 only. This contrasts with the CD56^br^ NK subset that expands *in vitro* in the presence of IL-2 stimulation. These differences underscore the importance of stratifying the CD56^br^ and CD56^dim^ NK cell subsets in blood to understand the specific contribution of each subset. The distinct proliferative capacities of these two NK cell subsets can lead to erroneous conclusions when culturing bulk NK cell populations, due to the strong preferential survival of the CD56^br^ NK cell subset *in vitro*, which can easily become predominant after a few days in culture. Further to the specific changes in the NK cell subsets, our data also demonstrate the relevance of limiting the functional heterogeneity of the target cell population to reduce the variability of *in vitro* NK co-culture assays. In this study we show that CD4^+^ Tconvs and Tregs also display distinct dynamics of response to αCD3/CD28 stimulation, leading to the induction of different proliferative rates. These differences highlight the need to carefully normalize assay conditions to limit the variability associated with changes in the cell’s proliferative state. These considerations are particularly relevant when directly comparing CD4^+^ Tconvs and Tregs using *in vitro* functional assays, as they can lead to changes in the assay readout due to specific differences in proliferation rates.

Recently, we have characterized the changes in CD56^br^ NK cells in patients treated with LD-IL-2 using a single-cell multiomics approach [19]. Notably, we observed increased proliferative capacity and activated phenotype in CD56^br^ NK cells after a month-long treatment period with a 3-day dosing interval schedule. The finding that CD56^br^ NK cells kill proliferating Tconv and Tregs similarly could entail the detrimental preferential killing of the expanded Treg subset in patients treated with LD-IL-2. However, in the context of LD-IL-2 immunotherapy, the expanded Tregs in blood are unlikely to be acutely activated, and therefore not susceptible to killing by CD56^br^ NK cells. A more plausible mechanism is that CD56^br^ NK cells activated in response to LD-IL-2 treatment rapidly migrate to tissues where they could exert an immunoregulatory function in the elimination of chronically activated T cells, thereby providing a rationale for the clinical benefit observed in patients undergoing LD-IL-2 immunotherapy [26–30]. This could be relevant not only in the context of autoimmune diseases, by targeting autoreactive T cells expanded at the sites of inflammation, but also in the context of potentially detrimental anti-tumour responses, by eliminating hyperproliferative effector T cells. We also note that although we did not observe a preferential killing of proliferating T cells within the broader CD56^dim^ NK subset, the very poor proliferative potential and survival of these cells in our *in vitro* assay conditions may significantly reduce our power to identify these changes. One consistent observation stemming from LD-IL-2 immunotherapy studies [16–19], is that even very low amounts of IL-2 can significantly expand the proportion and numbers of CD56^br^ NK cells, but not CD56^dim^ NK cells, in blood. The original observation in the non-obese diabetic (NOD) mouse model of autoimmune diabetes that activated NK cells infiltrating the pancreatic islets were involved in the pathogenesis of the disease [31, 32] raised questions over their putative detrimental role in the human disease. These findings led pharmaceutical companies to explore the development of IL-2 mutein molecules characterized by a reduced capacity to elicit NK cell proliferation [33, 34]. However, the recent promise of LD-IL-2 trials in several autoimmune and inflammatory diseases [20, 21] demonstrates the need to better understand the function of the IL-2-expanded CD56^br^ NK cells and their specific contribution to the drug’s mechanism of action. Given the technical limitations with functional *in vitro* NK killing assays, it is critical to develop better tools to investigate these cells in clinical samples from completed and ongoing trials.

Our current study presents several limitations to consider. The first limitation is that we did not define the exact molecular mechanism underlying the preferential killing of proliferating T cells by CD56^br^ NK cells. It is likely that some of the mechanisms used by CD56^br^ NK cells to kill activated T cells [25] are acting preferentially on the proliferating cells. The activation of T cells *in vitro* with αCD3/CD28 stimulation induces a vast number of changes to the transcriptional profile of T cells, and therefore there are a variety of targets that may affect the dynamics of NK:T cell interaction and mediate the preferential targeting of proliferative cells. Furthermore, it is also plausible that αCD3/CD28 stimulation induces the upregulation of pro-apoptotic responses to limit overt T cell activation and effector T cell expansion [35], thereby leading to increased susceptibility to NK cell killing. Further work is therefore warranted to investigate the mechanism of NK-cell mediated killing of proliferative T cells and how it could potentially be modulated *in vivo*. Another limitation of our study was the relatively small change of ~1.5-fold increased killing efficiency in proliferating T cells. It is difficult to estimate the exact physiological impact of these observed changes in our *in vitro* assay, and how they could translate to a much more complex system *in vivo*. Relatively small changes (<2-fold) have been previously shown to lead to robust and quantifiable phenotypic alterations. For example, we have previously shown that a genetic variant in the *IL2* locus provided only a 2-fold increased expression of IL-2 in the NOD mouse model, which was associated with a robust 80% increased survival [36]. It is notable that in organisms with long life span, such modest effects can have significant cumulative effects over time, thereby contributing to much greater biological implications. This is particularly relevant in a setting such as life-long NK cell-mediated protection from potentially pathogenic proliferative T effector cells. Finally, a third limitation is the high cell number requirement for the *in vitro* NK killing assay described in this study. This requirement is particularly relevant in the context of clinical studies, which, in its current format, may preclude the application of the assay to limited cryopreserved clinical samples.

Our observation that CD56^br^ NK cells kill proliferating T cells more efficiently lends support to a putative regulatory function of this subset *in vivo* by eliminating proliferative T cells in the context of an inflammatory response in the tissues. This may be a key regulatory mechanism to control overt T effector cell proliferation and activation in response to local IL-2 production at the sites of inflammation and is consistent with the higher proportion of CD56^br^ NK cells in tissues compared to circulation [37]. A regulatory role for CD56^br^ NK cells has been previously suggested in the context of multiple sclerosis patients treated with anti-CD25 (daclizumab) [38, 39]. Response to treatment in these patients was associated with a significant expansion of CD56^br^ NK cells and concomitant decline in the frequency of both CD4^+^ and CD8^+^ T cells in blood, thereby supporting a regulatory role of CD56^br^ NK cells via NK cell-mediated negative immunoregulation of activated T cells. Furthermore, CD56^br^ NK cells have previously been shown to be able to recognize and kill early activated T cells [6], which together with the observations that CD56^br^ NK cells kill tumour cells [40] and increased proportions of CD56^br^ NK cells in blood are associated with better prognosis in several types of tumours [40–42], warrants further work to understand how they may contribute to the regulation of anti-tumour responses. Taken together, these findings support a physiological regulatory role of CD56^br^ NK cells in an inflammatory context. Our results raise the possibility that CD56^br^ NK cell expansion by low-dose IL-2 is a positive feature and could contribute to the previous reports of clinical efficacy in diverse inflammatory conditions [26–30].

## Data Availability

The code and data used for the statistical analyses are availabe at https://github.com/arcadio/nk-models.

## Abbreviations:

BV: Brilliant Violet™
BUV: Brilliant™ Ultraviolet
CD56^br^: CD56^bright^ natural killer cell
CI: confidence interval
CV: coefficient of variation
FACS: fluorescence-activated cell sorting
IL-2: interleukin-2
IU: international units
LD-IL2: low-dose IL-2
NK: natural killer
Tconv: CD4^+^ conventional T cell
Treg: CD4^+^ regulatory T cell
